# Opportunity for natural selection among some selected population groups of Northeast India

**DOI:** 10.4103/0971-6866.69328

**Published:** 2010

**Authors:** Farida Ahmed Das, Sikdar Mithun

**Affiliations:** Department of Anthropology, Dibrugarh University, Dibrugarh, Assam - 786 004, India

**Keywords:** Fertility, mortality, Northeast India, selection intensity

## Abstract

**BACKGROUND:**

Selection potential based on differential fertility and mortality has been computed for seven population groups inhabiting different geographical locations of Northeast India.

**MATERIALS AND METHODS:**

Crow’s as well as Johnston and Kensinger’s index have been used for the present purpose.

**RESULTS AND CONCLUSION:**

Irrespective of the methodology, the total index of selection was found to be highest among the Deoris followed by the Kaibartas. The lowest selection index was found among the Oraon population. If the relative contribution of fertility and mortality components to the total index is considered to be multiplicative, it is observed that in all these communities the fertility component exceeds that of mortality component, which may indicate initiation of demographic transitional phase in the selected populations with the betterment of healthcare and socioeconomic condition within the last few decades.

## Introduction

Natural selection is one of the major evolutionary forces that bring about changes in the gene frequencies in a population through the action of differential fertility and mortality over generations. Differential fertility and mortality are the basic events of natural selection, which operate singly or jointly to determine the fitness of a particular population in a given environment.[1] Crow[2] devised an index that facilitates quantitative estimation of the selective pressure, given the reproductive pattern of a population. This is a generation analog of Fisher’s[3] ‘Fundamental Theorem of Natural Selection’ and measures the proportion by which fitness would increase with specific birth and death rates if they were all selective and the heritability of fitness were complete.[4] But, in practice, the genetic component in differential fertility and mortality is not the sole factor, as the reproductive outcome of an individual and or/population is a result of the interaction of variety of sociocultural factors that affect the fertility and mortality behavior of a particular population. Thus, the index only sets an upper limit for the potential action of natural selection and is accordingly renamed as opportunity for natural selection.[5] It can be divided into two components, i.e., selection due to differential fertility and selection due to differential mortality. It is more descriptive than analytical[4] and can lay out the facts that can be derived from vital statistics. Though several authors have pointed out the difficulties in using and interpreting the index,[6,7] Crow’s index is considered to be one of the most feasible means of inferring selection as it is difficult to measure selection directly in human population. As such this is the index mostly used worldwide. Later, the index was modified by Johnston and Kensinger[8] to include prenatal mortality.

In the present study, the total index of natural selection is worked out to focus on the opportunity of natural selection among seven endogamous population groupsof Northeast India. The population groups thus studied have different ethnocultural histories and have different socioeconomic backgrounds.

## Material and Methods

The materials for this study consisted of 196 women belonging to seven different ethnic communities of Northeast India. Seven villages, where the studied population groups live homogenously, were visited. A detailed demographic account of the investigated villages was collected. A total of 72 Deori, 27 Manipuri Meities, 24 Garo, 19 Oraon, 15 Kaibarta, 16 Assamese Muslim, 23 Tai-Khamyang evermarried women who had completed their fertility period were interviewed to get the relevant information for this study. An interview schedule was designed and was used to get the detailed reproductive history of these women. The information was later checked with their husbands and elderly members of the family. Crow’s[2] as well as Johnston and Kensinger’s[8] indices were applied to find out the opportunity for natural selection. The formulae that are used are shown below.

Crow’s index can be computed by using his formula (Crow, 1958) and the modified one that includes the prenatal losses (Johnston and Kensinger, 1971). The computed procedures are given below.

Crow’s index:

$$I\;=\;I_{m}+\;I_{f}/P_{s}$$

$$I_{m}\;=\;P_{d}/P_{s}$$

$$P_{s}\;=\;1/P_{d}$$

$$I_{f}\;=\;V_{f}/X^{2}$$

where *I* is the index of total selection intensity, *I*_m_ the index of selection due to mortality, *P*_d_ the probability of deaths up to prereproductive age, *P*_s_ the probability of survival upto reproductive age, *I*_f_ the index of selection due to fertility, *V* the variance due to fertility, and *X* is the mean number of live births.

Johnston and Kensinger’s index:

$$I\;=\;I_{\mathrm{me}}\;+\;I_{\mathrm{mc}}/P_{b}\;+\;I_{f}/P_{b}\;\times \;P_{s}$$

$$I_{\mathrm{me}}\;=\;P_{\mathrm{ed}}/P_{b}$$

$$P_{b}\;=\;1\;-\;P_{\mathrm{ed}}$$

$$I_{\mathrm{mc}}\;=\;P_{d}/P_{s}$$

$$P_{s}\;=\;1\;-\;P_{d}$$

$$I_{f}\;=\;V/X^{2}$$

where *I* is the index of total selection intensity, *I*_me_ the index of total selection due to prenatal mortality, *P*_ed_ the probability to die before birth, *P*_b_ the probability to survive till birth, *I*_mc_ the index of total selection due to postnatal mortality, *P*_d_ the probability to die before reaching reproductive years, *P*_s_ the probability to survive till reproductive age, *I*_f_ the index of selection due to fertility, *V* the variance due to fertility, and *X* is the mean number of live births. *P*_d_ and *P*_s_ are calculated based on prereproductive deaths.

A brief descriptive account of the studied population groups has been provided below.

### Deoris

The Deoris constitute one of the distinct tribes of Assam. Linguistically, they belong to the Tibeto-Burman linguistic family of Mongoloid stock. There are four great territorial groups or *Khels* among the Deoris, viz., Tengapania, Borgonya, Dibongia, and Patorgonya. Each one derived its name either from a particular place or river. The last group has reportedly become extinct. Each of these divisions is again divided into a number of exogamous clans *Bansha* or *Phoidya*. Each group has certain distinctive features which help it to maintain its own identity. Nowadays, the Deoris are found in North Lakhimpur, Dhemaji, Tinsukia, Sibsagar, Jorhat, and Sonitpur districts in Assam, while in Arunachal Pradesh they are found in places like Mahadevpur, Tezu, Namsai, Sumpoi, Mohong, Dharampur, etc. The Deoris are Hindus by religion and the followers of *Balia Baba*, an incarnation of Lord Shiva. This study had been conducted in Mahadevpur Deori village situated in the eastern side of Namsai, the head quarter of Lohit subdivision, Arunachal Pradesh. The villagers belong to the Tengapania group of Deoris.

### Manipuri Meities

The Meities are predominantly settled in the central valley of Manipur, a small state of India situated in the northeastern corner of the country. Their settlements have also been reported from other states of India like Assam, Tripura, and Uttar Pradesh.[9] The Meities are described as the Kuki-chin section of the Tibeto-Burman stock by Chatterjee.[10] Though the general features of the Meities are Mongoloid, there has been a great diversity of features among them, some of which approach Aryan type.[11] The data from this population group were collected from Borkula Hanchara village under Sibsagar district, Assam. The village is homogeneously inhabited by Meities only.

### Garo

The Garo is a major matrilineal tribal population of Northeast India inhabiting mostly the Garo hills district of Meghalaya. A small section of them is also found to be scattered in different places of Assam. They call themselves as ‘Achik-mande’ which literally means ‘Hill man’. Ethnically, the Garos belong to the Bodo group of Tibeto Burman linguistic family. They call their dialect ‘achik kusik’ which literally means ‘voice of the hills’. Data for the present investigation were drawn from Poschim Bosti, a village homogenously inhabited by Garo people. They were generally brought into the present village by the British for various reasons, one of which was for cutting the forests for tea gardening. The people of the village are Christians by religion and cultivation is their main source of livelihood.

### Oraon

The Oraon is a Dravidian speaking tribe. According to some authorities, the tribe is broadly divided into five subtribes and they are known as Berga Oraon, Dhanka Oraon, Kharia Oraon, Khendro Oraon, and Munda Oraon. Besides, there are a number of exogamous clans in each group. At present, their principal settlement is Ranchi district of Jharkhand. They are also found in Bihar, Madhya Pradesh, Orissa, West Bengal, and Assam. In Assam, the Oraons were brought by the British as a labor group for the tea gardens. Now they constitute one of the major tea garden labor populations in Assam. In Assam, they used to call themselves as ‘Urang’. Most of them are attached to the tea industry but with the passage of time they are also found to be attached to various other livelihoods. The data for the present work were collected from Tingkhong Tea Estate of Dibrugarh District, Assam. The work was confined to an area inhabited by Oraon community only.

### Kaibarta

The Kaibarta is a scheduled caste population of Assam. They prefer to live on the river banks or near the sources of water where fishes are available. Their main traditional occupation is fishing. Though cultivation of paddy is also a subsidiary source of livelihood, they prefer their traditional occupation. Most of them earn their livelihood by selling fish. Womenfolk take active part in their traditional occupation. In the Assamese Hindu society, the Kaibarta occupies the lowest position in the caste hierarchy. This study was conducted in the Uppor Nazira Kaibarta village under Sibsagar district of Assam, which is 12 km away from Sibsagar town.

### Assamese Muslims

In Assam, Muslims constitutes the second largest of the mass. As per the historic records, the present day Assamese Muslims are the descendents of three different Muslim groups.[12] One of them is known as ‘Garia’, as they claim to have come from Gaur, the ancient Muhammadan capital of Bengal.[13,14] The second one is known as ‘Maria’ who are said to be the descendents of prisoners captured when Turbok, a pathan general, was defeated and killed in 1532 AD.[15] The third section is known as ‘Sayeds’ or ‘Daon’, who trace their ancestry from Azan Fakir, a 17^th^ century Muslim saint who came from Baghdad. All the three sets form endogamous groups among themselves and prefer to marry among themselves. The people for this present study belonged to the Garia sect and were concentrated homogeneously in the Uppor Nazira Muslim village under Sibsagar district. The village is 12 km away from Sibsagar town.

### Tai-Khamyang

Tai group is one of the significant elements that entered Assam in the historic past. The Khamyangs or Khamjangs who are popularly known as ‘Shyams’ is a section of the great Thai or Tai-stock. They had their independent principality in Mungkong till the end of the 18^th^ century. Mung means ‘a country’ and Kong means ‘a special king of drum’ called Dhakdhol. Linguistically, they belong to the Tai-speaking group and they are Buddhists of the Teravada school of religion. Earlier, they used to interact among themselves through Tai or Pali dialect. But later, when they came into contact with the local autochthons after migrating from their original abode, they gradually adopted Assamese as their language for conversation. Though they reside with the neighboring people, they still preserve their traditional cultural peculiarities like dress pattern, food and drink, fairs and festivals, clan organization, etc. This study was conducted in the Cholapathar village in Sibsagar district. Agriculture is their main source of livelihood; however, they are also found to engage themselves in other public as well as private jobs.

## Results

The mean number of live births along with its variance and other relevant parameters to detect the selection intensity among the seven population groups is shown in Table 1. It is evident from the table that the Oraons have the highest live births (7.58) while the Deoris have the least (4.38) live births of all the seven studied populations of Northeast India. The variance of live births is found to be the highest among the Oraons (4.97) and it is followed by the Meities (3.99). The least variance of live births is found among the Assamese Muslims. The intensity of natural selection in terms of Crow’s index is found to be the highest among the Deoris (0.445) and the lowest among the Oraon population (0.176) [Table 2]. The Meities, Garos, Kaibartas, Assamese Muslims, and Tai-Khamiyang people share relatively similar selection pressure which is found to be 0.027, 0.244, 0.267, 0.232, and 0.246, respectively. The postnatal mortality index is found to be the highest among the Deoris (0.226) and lowest among the Assamese Muslim population (0.075). The fertility index is found to be the highest among the Deoris (0.179) and lowest among the Oraons (0.087). The indices of selection intensity as per Johnston and Kensinger in the seven populations have been shown in Table 3. It also indicates a pattern similar to that of Crow’s index in the relative occurrence of selection pressure in different population groups. The highest opportunity of natural selection is found among the Deoris (0.546) and the lowest among the Oraons (0.266). The embryonic mortality index is found to be highest among the Oraons (0.076) while it is lowest among the Tai-Khamyang population (0.028). The relative percentages of fertility and mortality components to the total index have been evaluated on the basis of Johnston and Kensinger’s index and have been shown in Table 4. The percentage of fertility component is found to be higher than that of the mortality component in all the population groups considered. The fertility component is found to be the highest among the Assamese Muslims (57.97%) and the lowest among the Manipuri Meities (42.75%). On the other hand, the percentage of mortality component to the total index is found to be the highest among the Deoris (41.40%) and the lowest among the Assamese Muslim population (0.075%).

**Table 1:** Parameters used in calculating opportunity for natural selection

Population	*X*	Variance	*P*_ed_	*P*_b_	*P*_s_	*P*_d_	*P*_b_*P*_s_
Deori	4.38	3.42	0.07	0.93	0.82	0.18	0.76
Manipuri Meities	6.30	3.99	0.05	0.95	0.91	0.09	0.87
Garo	5.21	3.09	0.03	0.97	0.90	0.10	0.87
Oraon	7.58	4.97	0.07	0.93	0.92	0.08	0.86
Kaibarta	5.33	3.53	0.06	0.94	0.89	0.11	0.84
Assamese Muslim	4.50	2.95	0.04	0.96	0.93	0.07	0.89
Tai-Khamiyang	4.65	3.06	0.03	0.97	0.92	0.08	0.89

**Table 2:** Crow’s index for opportunity of natural selection in different population groups of Assam

Population	*I*_m_	*I*_f_	*I*_f_*IP*_s_	*I*
Deori	0.226	0.179	0.219	0.445
Manipuri Meities	0.097	0.101	0.111	0.207
Garo	0.116	0.114	0.127	0.244
Oraon	0.083	0.087	0.094	0.176
Kaibarta	0.127	0.124	0.140	0.267
Assamese Muslim	0.075	0.146	0.157	0.232
Tai-Khamiyang	0.092	0.142	0.154	0.246

**Table 3:** Johnston and Kensinger’s index of opportunity for natural selection

	*I*_me_	*I*_mc_	*I*_f_	*I*_f_*IP*_b_*P*_s_	I
Deori	0.070	0.226	0.179	0.234	0.546
Manipuri Meities	0.053	0.097	0.101	0.116	0.271
Garo	0.032	0.116	0.114	0.132	0.283
Oraon	0.076	0.083	0.087	0.101	0.266
Kaibarta	0.063	0.127	0.124	0.149	0.346
Assamese Muslim	0.042	0.075	0.146	0.164	0.283
Tai-Khamiyang	0.028	0.092	0.142	0.159	0.281

**Table 4:** Percentage of fertility and mortality components of the total index (based on Johnston and Kensinger’s index)

Population	Percentage of fertility component	Percentage of postnatal mortality component
Deori	42.87	41.40
Manipuri Meities	42.75	35.75
Garo	46.59	40.94
Oraon	37.92	31.16
Kaibarta	43.07	36.71
Assamese Muslim	57.97	26.51
Tai-Khamiyang	56.52	32.70

The intergroup comparisons for embryonic and postnatal mortality estimates based on standardized normal deviate[16] have been shown in Tables 5 and 6, respectively. It is evident from the tables that Oraon population varies significantly with Deoris, Garos, Assamese Muslims, and Tai-Khamyangs, with respect to embryonic mortality. The intergroup differences are, however, not significant among the other populations. *Z* score values for intergroup variability with regard to postnatal mortality reveal that the Deori population varies significantly with all the populations except Kaibartas while the differences are not significant between the other population groups.

**Table 5:** Intergroup comparison for embryonic mortality estimates based on standardized normal deviate (*Z*)

Population	Manipuri Meities	Garo	Oraon	Kaibarta	Assamese Muslim	Tai-Khamiyang
Deori	0.265	1.325	2.204*	0.211	0.947	1.660
Manipuri		1.363	1.654	0.000	1.030	1.674
Meities						
Garo			2.816*	1.201	0.169	0.348
Oraon				1.424	2.354*	3.069*
Kaibarta					0.927	1.499
Assamese						0.485
Muslim						

*Z* = (*X*_1_/*n*_1_) – (*X*_2_/*n*_2_)/√*p* (1 – *p*) (1/*n*_1_ + 1/*n*_2_), where *X*_1_ and *X*_2_ are the characteristics ‘A’ in *n*_1_ and *n*_2_ samples of the population N1 and N2, respectively, and *P* = (*x*_1_ + *x*_2_)/(*n*_1_ + *n*_2_);

* Significant at 5% level

**Table 6:** Intergroup comparison for postnatal mortality estimates based on standardized normal deviate (Z)

Population	Manipuri Meities	Garo	Oraon	Kaibarta	Assamese Muslim	Tai-Khamiyang
Deori	2.517*	2.551*	2.052*	1.721	3.955*	3.793*
Manipuri		0.099	0.157	0.278	1.544	1.158
Meities						
Garo			0.244	0.357	1.427	1.032
Oraon				0.123	1.576	1.211
Kaibarta					1.607	1.259
Assamese						1.607
Muslim						

*Z* = (*X*_1_/*n*_1_) – (*X*_2_/*n*_2_)/√*p* (1 – *p*) (1/*n*_1_ + 1/*n*_2_), where *X*_1_ and *X*_2_ are the characteristics ‘A’ in *n*_1_ and *n*_2_ samples of the population N1 and N2, respectively, and *P* = (*x*_1_ + *x*_2_)/(*n*_1_ + *n*_2_);

* Significant at 5% level

## Discussion

The computed mortality and fertility components and the index of the total selection intensity as per Crow (1958) indicate a relatively low index of total selection among the studied populations of Northeast India. Recently, Reddy and Chopra[17] have reported that selection index (*I*) in the Indian population ranges between 0.258 and 2.250. Thus, the present findings are inclined more toward the lower limit of Indian range. This finding goes in conformity with several other studies done recently in different parts of India.[18–22] As far as the contribution of fertility and mortality components to the total index (Johnston and Kensinger) is concerned, it is observed that fertility component contributes more to the total index than the mortality component in all the population groups. A similar observation has also been made by Sikdar[23] among the other different population groups of Northeast India in recent years. Reddy and Chopra,[17] however, found this trend only among 34% of the population groups out of 96 populations considered for the purpose. The Deori and the Oraon population present a high index of postnatal mortality and prenatal mortality, respectively. It is also found to be responsible for the significant intergroup variation for postnatal mortality estimates of Deoris and prenatal mortality estimates of Oraons with the other population groups. A relatively high prenatal and postnatal mortality among these two population groups may be because of relatively poor medical and healthcare facilities available in relation to the other studied groups. In this respect it can be noted that the Deori community studied for the present purpose resides near the hilly terrain of Arunachal Pradesh where the people can avail least amount of the medical facilities. As per Singh,[24] the incidence of child mortality greatly depends upon the social status of the particular population. Thus, the present findings may be influenced by the social status of the population groups concerned.

The study reveals a low intensity of natural selection among these seven population groups. Thus, the value of selection intensity is also relatively low in the studied populations in relation to the Indian ranges. Low values of selection intensity indicate a low pressure of natural selection in these populations. This may be because of the operation of different factors like sociocultural, better economic and education system, health awareness, and medical facilities among different population groups in the recent years. The low intensities of selection along with lower mortality indices may suggest that all these population groups are at the initial stage of demographic transition because it is suggested that at the initial stage of transitional phase selection index decline initially followed by a rapid decline of mortality component with stable fertility component (Sikdar)[23]. However existence of variability in the selection index within these populations reflects variability in the accessibility of various healthcare measures among them.
